# A model-independent approach to infer hierarchical codon substitution dynamics

**DOI:** 10.1186/1471-2105-11-201

**Published:** 2010-04-23

**Authors:** Olof Görnerup, Martin Nilsson Jacobi

**Affiliations:** 1 Complex Systems Group, Department of Energy and Environment, Chalmers University of Technology, 412 96 Göteborg, Sweden

## Abstract

**Background:**

Codon substitution constitutes a fundamental process in molecular biology that has been studied extensively. However, prior studies rely on various assumptions, e.g. regarding the relevance of specific biochemical properties, or on conservation criteria for defining substitution groups. Ideally, one would instead like to analyze the substitution process in terms of raw dynamics, independently of underlying system specifics. In this paper we propose a method for doing this by identifying groups of codons and amino acids such that these groups imply closed dynamics. The approach relies on recently developed spectral and agglomerative techniques for identifying hierarchical organization in dynamical systems.

**Results:**

We have applied the techniques on an empirically derived Markov model of the codon substitution process that is provided in the literature. Without system specific knowledge of the substitution process, the techniques manage to "blindly" identify multiple levels of dynamics; from amino acid substitutions (via the standard genetic code) to higher order dynamics on the level of amino acid groups. We hypothesize that the acquired groups reflect earlier versions of the genetic code.

**Conclusions:**

The results demonstrate the applicability of the techniques. Due to their generality, we believe that they can be used to coarse grain and identify hierarchical organization in a broad range of other biological systems and processes, such as protein interaction networks, genetic regulatory networks and food webs.

## Background

Ever since its discovery by Nirenberg and Matthaei [1], the structure [2-8] and evolution [9-18] of the genetic code from nucleotide triplets in DNA to amino acid residues in proteins has been studied extensively. In structure-based studies--in terms of a snapshot of what codes to what--similar codons have for instance been found to be associated with amino acids with similar properties [19] and amino acids with simple structures are typically coded by more codons [6]. However, amino acids may be grouped with respect to several different properties, and it is difficult to quantitatively judge the relative and actual relevance of these properties. By studying the effective evolutionary dynamics of codons and amino acids one avoids this problem. In dynamic-based approaches the substitution process is often modeled as a Markov chain, where the distribution of substitutions of a given residue is independent of neighboring residues as well as prior residues at the same site. These assumptions are clearly not strictly correct, but are still meaningful as approximations. Dayhoff and coworkers pioneered Markov modeling by estimating substitution frequencies empirically from alignments of orthologous sequences [20]. From inspection of log odds scores they concluded that amino acids with similar properties indeed tend to form groups that are conserved. In other words, members of a group usually substitute to each other, rather than to external residues. In subsequent work [21-24], conservation has been turned into a criteria used for defining and inferring amino acid substitution groups. An interpretation of these results is that the substitution process hierarchically operates on multiple levels, from nucleotides to codons to groups of codons [4]. However, one relies on strong assumptions when aiming to infer hierarchical levels in terms of certain biochemical properties or explicit conservation criteria. Ideally, one would instead like to "blindly" infer levels purely from the observed dynamics. In this paper we present such an approach, which is based on recently developed methods for identifying hierarchical levels in dynamical systems [25]. The methods are derived from first principles, and only rely on the assumption that the dynamic process can be described as a Markov chain; there are no assumptions regarding for example amino acid conservation or group isolation. In fact, the techniques presented here are not limited to the substitution process, but may also be applied to the broad range of biological systems that can be represented by networks or transition matrices. In this presentation, however, we will concentrate on the substitution process by applying the techniques on an empirically derived codon transition matrix provided by Schneider et al. [26].

In the next section we will present our methodology. The underlying theory is only introduced briefly here, and will have a focus on Markov chains. A more thorough presentation (including proofs) that covers a broader class of systems can be found in Ref. [25]. We will then report on the results when applying our techniques on the codon transition matrix. After discussing the acquired results and their possible relation to the evolution of the genetic code, we conclude the paper with a few closing remarks about the methods relation to biological modeling in general, and possible future directions.

## Methods

**Figure 1:**
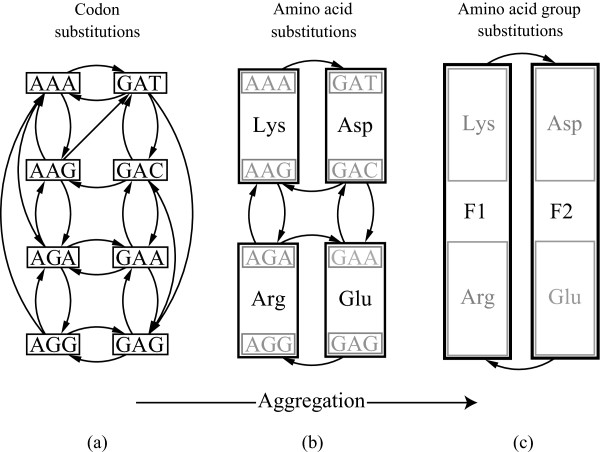
**Three levels of dynamics by Markov chain lumping**. (a) Codon substitutions are modeled as a Markov chain. States represent codons and transitions represent substitutions between codons. (b) If the codons are aggregated with respect to the amino acids they code for, the new aggregated process remains a Markov chain. For instance, since AAA and AAG both code for lysine, they can be aggregated into one unit. (c) Specific aggregates of amino acids also exist such that their dynamics is Markovian. Lysine and arginine can for example be merged to form one state.

Hierarchical organization is an intrinsic property of complex systems as it is a prerequisite for a system to stably evolve complexity [27]. Formally, a hierarchy can be viewed as a set of levels at which the system operates, where each level approximately has its own closed dynamics. Each level is defined by an aggregation (grouping) of states. Aggregating a Markov chain, which we consider here, means that the state space is partitioned into macro-states. The original dynamics and the partition of the state space then defines a new stochastic process on the coarser level. However, in general such an aggregation does not define a proper level of description in the hierarchy since the partition introduces memory on the aggregated level. Put differently, the dynamics on the aggregated level is not closed. In the special case when the aggregated dynamics indeed is closed, the stochastic process over the partitions constitutes a Markov chain with the same order as the original process. In such cases, the aggregation is termed *lumping *in the literature, and the Markov chain is said to be *lumpable *[28]. See Figure 1 for a schematic illustration of Markov chain lumping in the context of the codon substitution process.

The degree by which a coarser process fulfills the Markov criteria (i.e. its degree of closeness) can be measured for example as the expected mutual information, denoted ⟨I⟩, between the process' past and future states, given its current state. Let {*s*_1_, *s*_2_, ..., *s*_*n*_} be the state space of an aggregated process, *P*_*i *_a stochastic variable of the preceding state of *s*_*i*_, and *F*_*i *_a stochastic variable of the subsequent state of *s*_*i *_(here we only consider one step--in general the past and future may be of arbitrary length). The mutual information between past and future states, given a current state *s*_*i *_is(1)

where H(*P*_*i*_) is the Shannon entropy(2)

of *P*_*i*_. H(*F*_*i*_) and H(*P*_*i*_, *F*_*i*_) of the joint distribution of *P*_*i *_and *F*_*i *_are defined analogously. Then(3)

where Pr(*s*_*i*_) is probability that the system is in state *s*_*i*_. The criterion can be used to test whether or not a given partition defines a lumping, but it is typically not useful for constructing the partitions that define lumpings. Since the number of possible ways to partition a state space of *N *states is astronomical even for relatively small *N *it is not feasible to evaluate all partitions. Instead, we employ two novel techniques for identifying aggregations that enables one to analyze systems with a large number of states (on the order of 10^3^, or 10^4 ^if the transition matrix is sparse). The first technique is based on the following observation (see [25,29] for further details): Consider *n *eigenvectors of a transition matrix. These will define *N *points in an *n*-dimensional space, where each point is associated with a state in the Markov chain. *If the N points form n clusters, these clusters define an aggregation, where aggregates of states are given by corresponding points within clusters*. The task of finding aggregations is then reduced to the problem of finding *n *eigenvectors that respect the same *n *clusters of eigenvector elements. We will illustrate this with an example. Consider a Markov chain whose dynamics over some state space {*a, b, c, d*} is given by a transition matrix(4)

where *p *and *q *are probabilities. We can determine if the states can be aggregated by examining *P*'s eigenvectors. These are given by the columns in(5)

where *r *= *p*(*q *- 1)/*q *and . We see that there are two clusters in the second eigenvector (with values 1 and -1). Since the first eigenvector respects the same clusters (trivially so since the first eigenvector forms a single cluster), the first and the second eigenvectors define an aggregation, namely {{*a, c*}, {*b, d*}}. There are also two trivial aggregations: {{*a, b, c, d*}} (due to the first eigenvector alone) and {{*a*}, {*b*}, {*c*}, {*d*}} (due to all eigenvectors, assuming they form a complete base). Due to the conservation of probability in a Markov process, the trivial aggregations where all states are in the same aggregate always exist.

Identifying *n *eigenvector constitutes a constraint satisfaction (SAT) problem. We have implemented a backtracking algorithm that in the typical case identifies aggregates in polynomial time. It is beyond the scope of this paper to describe the algorithm here. Instead we refer to [30], where we specify the algorithm in detail and provide pseudocode.

The spectral method works best for inferring large aggregates, but in order for small aggregates to be identified, they need to be distinct. Therefore we also use a second technique akin to agglomerative clustering. It works in the following:

1. Initialize an aggregation  as the partition where each partition element consists of one element.

2. Evaluate every partition where two elements of  are merged by calculating the expected mutual information hIi (Eq. 3) (there are ||(||-1)/2 partitions to test).

3. Let ℬ be the partition that resulted in the lowest ⟨I⟩.

4. Replace  with ℬ and repeat from step 2.

That is, initially each state is in a separate partition element, and the state space is then successively aggregated by joining the pair of aggregates that result in the lowest mutual information. The agglomeration method gives good results on the first levels in the aggregation hierarchy, but becomes less accurate at coarser levels. Since the spectral method works best in the latter case, the techniques complement each other.

We have applied the two techniques on a Markov chain of codon substitutions, whose transition probabilities have been empirically derived by Schneider et al. [26]. The codon substitution frequencies have been estimated from 17,502 pairwise alignments of orthologous sequences from human, mouse, chicken, frog and zebrafish. For this purpose, they aligned 8.3 million codons, counted the substitutions between codons, and derived the substitution probability matrix from the resulting counts.

## Results

**Figure 2:**
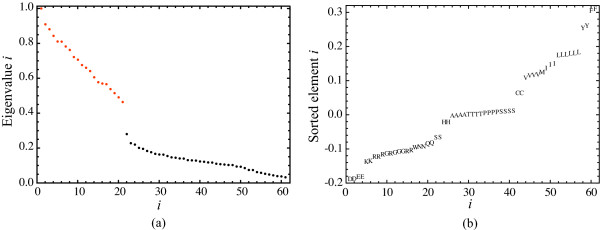
**A spectral gap and level sets reveal the genetic code**. Eigenvalues of the codon substitution transition matrix *P *(note that the system has 61 states as substitutions from the three stop codons are not considered). A distinct spectral gap after the 21st first eigenvalues (marked in red) suggests that the 21 first eigenvectors reveal an aggregation. (b) Vector elements of the fourth eigenvector of *P *are organized in level sets, where codons that map to the same amino acid are on the same level (with the exception of serine). All the eigenvalues are real because the transition matrix *P *is reversible.

A first observation is that the spectrum of the transition matrix provided by Schneider et al. has a clear gap after the 21st eigenvalue, Figure 2(a). This gap indicates a time scale separation and that the 21 first eigenvectors may reveal an aggregation of the substitution process. By clustering the elements of the 21 first eigenvectors of *P*-- resulting in 61 points in a 21 dimensional space--21 distinct clusters are acquired. Since the number of eigenvectors equals the number of clusters, these define a valid aggregation. As exemplified in Figure 2(b) the clusters show as level sets in the individual eigenvectors. The aggregation constitutes the standard genetic code as each cluster constitutes codons that are associated with the same amino acid, with the exception of the codons of serine, which are divided into two clusters ({TCT, TCC, TCA, TCG} and {AGT, AGC}). This unique separation is due to that serine is the only amino acid whose codons are not connected with single point mutations (i.e. some codons are separated by a Hamming distance larger than one on a hypercube).

**Figure 3:**
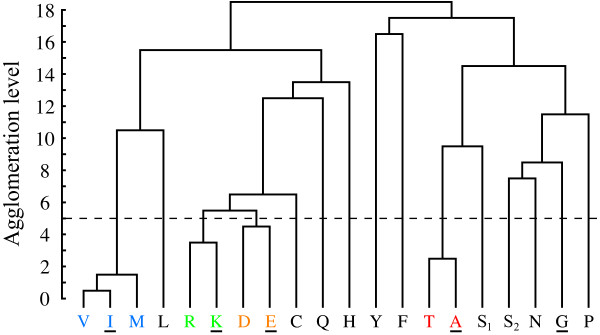
**Agglomeration progress**. A dendrogram of the result of an agglomeration based on successively joining pairs of states or aggregates that result in the best aggregate with respect to the mutual information measure in Eq. 3. The dashed line marks the most significant aggregation, which is also shown in Figure 4. S_1 _denotes serine coded by TCT, TCC, TCA and TCG, and S_2 _denotes serine coded by AGT and AGC. Amino acids forming Riddle et al.'s minimum set capable of forming complex protein folds [41] are underlined.

**Figure 4:**
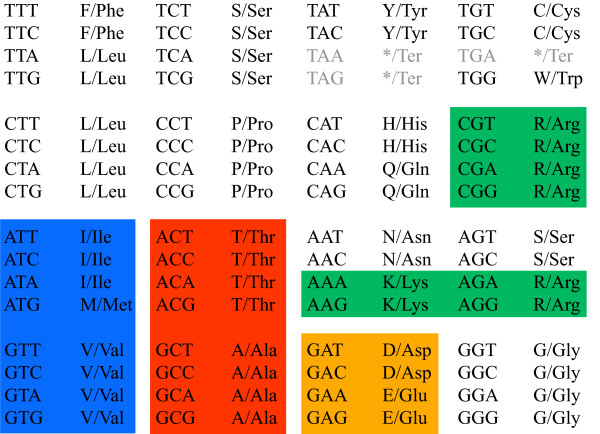
**The most significant amino acid aggregation**. Amino acid groups resulting in the most significant lumping {*A, T*}, {*I, M, V *}, {*E, D*} and {*K, R*} as shown in the standard genetic code table.

**Figure 5:**
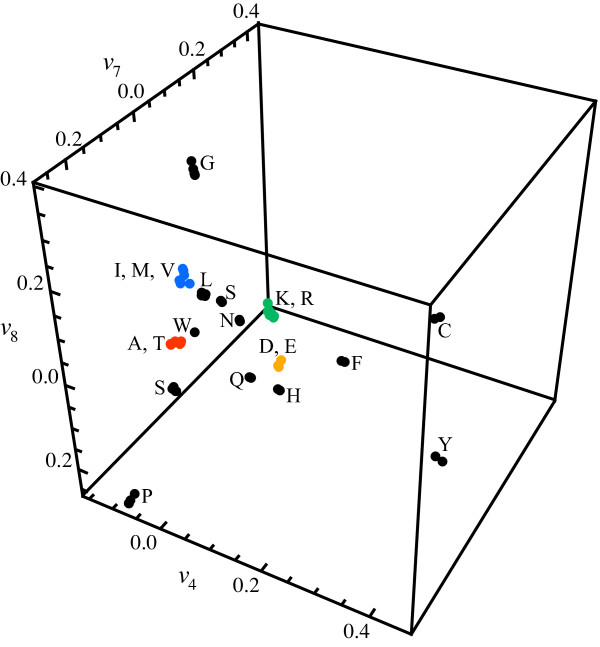
**Eigenvector clusters indicate aggregation levels**. Elements of the fourth, seventh and eighth eigenvector of the codon substitution matrix. Codons that map to the same amino acids are clustered, which indicate the standard genetic code. There are also clusters of amino acids in turn (marked with the same colors as in Figure 3 and 4): {*A, T*}, {*I, M, V*}, {*E, D*} and {*K, R*}, which indicate that these form higher order aggregates.

At the higher order aggregated level of amino acid substitution, lumpings are not as clearly revealed by the eigenvectors. This is expected since the redundancy in the genetic code reflects a much stronger neutrality than possible similarities between the amino acids. If the partitioning of the state space is viewed as an optimization problem aiming to minimize the mutual information defined in Eq. 3, then there are many almost equivalent minima. In this situation significant amino acid aggregates are identified by the complementary agglomeration technique. The progress of the procedure is shown in the dendrogram in Figure 3. Due to that tryptophan (W) has very low mutability and is the least occurring amino acid, a significant two-state lumping exists where tryptophan forms one aggregate and the rest of the amino acids form another aggregate. To simplify further analysis tryptophan is therefore discarded. The resulting most significant aggregation is given by {*A, T*}, {*I, M, V*}, {*E, D*} and {*K, R*}, cf. Figure 4. If we go back to the spectral view, we see that the same aggregation is indicated by three of the eigenvectors in the transition matrix, Figure 5. This exemplifies that one may also identify aggregates by searching for clusters or clear separations of eigenvector elements and then test if these constitute valid aggregates by using Eq. 3. For instance, one known grouping is to separate purine-centred and pyrimidine-centred codons [31,32]. Inspecting Figure 2(b) and Figure 5, we can see that the two groups indeed are separated if we exclude the rare amino acids *C*, *Y *and *W*, although not forming two distinct meta-clusters. However, a purine-pyrimidine separation is not present in the dendrogram in Figure 3.

## Discussion

We will now compare our results with amino acid groupings that previously have been discussed in the literature. Firstly, Jiménez-Montaño and He have used the same matrix that we employed here to perform hierarchical clustering of codons based on an Euclidian distance measure [33] (supplementary material). In their case *W *also forms its own aggregate and *S *is split up. Other similarities are the grouping of {*E, D*} and {*A, T*}. However, *A *and *T *are also grouped with *P*, *R *and *S*, and so their groupings do not respect the purine-pyrimidine separation. This is also the case in Figure 3, but not on the first aggregation levels. Another difference is that the genetic code is not as distinct in their case. The codons of *F*, for instance, are more separated than some of the amino acid aggregates (e.g. *A *and *T*).

Kosiol et al. [34] have estimated a different empirical codon model than the one used here and perform an aggregation with the Almost Invariant Sets (AIS) algorithm [35], which aims to find groups of elements that are conserved. They group codons into 20 and 7 aggregates and first identify the genetic code. In the 7-element aggregation, the aliphatics {*I, M, V, L*} form one group and the aromatics {*Y, F*} form one group (in our case, this is not as distinct, see Figure 3). Furthermore, half of the amino acids--those that are hydrophilic and basic--form one large group (in this way, *S *is not split). This group, however, does not respect the purine-pyrimidine separation. Kosiol et al. also apply the same algorithm on an empirical amino acid model [36] and acquire very similar results. One may argue that this is expected, since the AIS algorithm identifies the genetic code and since the aggregation of the codon model with respect to genetic code probably is very similar to the amino acid model. If we compare the aggregates acquired by Jiménez-Montaño et al. and Kosiol et al., we see that there is little agreement, with the exception that *A*, *S *and *T *are in the same aggregates in both cases.

Johnson and Overington have compared dendrograms based on twelve different scoring matrices with respect to a distance measure between scoring distributions [37]. In the resulting dendrograms all of our aggregates occur to various degrees (in 7, 4, 6 and 3 out of 12 times for {*K, R*}, {*A, T*}, {*E, D*} and {*I, M, V*}, respectively). Interestingly, all of our aggregates occur both in the dendrograms based on the scoring matrices by Gonnet et al [38] and Jones et al [39], where *W *also forms its own aggregate. Both these matrices are based on empirical sequence comparisons. In contrast, there is much less agreement with respect to matrices based on chemical or physical properties.

The standard genetic code is quite easily identified since member codons within an aggregate are invariant under mutations as they code for the same amino acid. It is not as clear, however, why the most significant amino acid aggregation is given by {*A, T*}, {*I, M, V *}, {*E, D*} and {*K, R*}--one of many other possible ways to group together amino acids. One may hypothesize that the aggregated dynamics of codon substitutions provide information about the origin of the genetic code. There are several theories aiming to address the fundamental question on how the code came to be. See Ref. [15] for a comprehensive comparison. With the exception of the *frozen accident *theory by Crick [40], these theories couple the evolution of the genetic code primarily with physico-chemical properties of the amino acids or evolved biosynthetic pathways. Woese [9], specifically, suggested that the code has evolved by a process of ambiguity reduction. The idea is that a crude primordial version of the code, where groups of codons code for groups of amino acids with resembling properties, evolved into the code's current state by a series of refinements. One may ask if amino acid groups reflect earlier versions of the code. Riddle et al. [41] experimentally searched for a minimum set of amino acids capable of forming complex protein folds. They found that the five amino acids *A*, *G*, *I*, *E *and *K *(underlined in Figure 3) are capable of forming most of the ancient SH3 protein domain. Consider again the most significant amino acid aggregation and note that *A*, *I*, *E *and *K *are all members of separate aggregates and that *G *forms its own aggregate. One could speculate that the aggregates reflect group codons in an earlier version of the code, and that these groups were specialized into present day codons. It is an intriguing hypothesis that is also partly supported by Jiménez-Montaño's hypothesis on the evolutionary history of the code [14]. In the proposed evolutionary tree, which is based on group theory and the thermodynamics of codon-anticodon interactions, amino acids within aggregates {*A, T*}, {*I, M, V*} and {*E, D*} share the same branches up till the two last reassignment of codons, although *K *and *R *are separated earlier than that (four reassignments). However, these observations remain speculative and clearly a more careful analysis would be required in order to conclusively relate acquired aggregates to the evolution of the standard genetic code and its deviates.

## Conclusions

We have employed two techniques to identify multiple levels of substitution dynamics. The techniques only consider the raw dynamics of the system and are therefore independent of system dependent assumptions that may be irrelevant or even false. The techniques therefore manage to blindly identify the amino substitution process via the standard genetic code, as well as higher order substitution dynamics via amino acid groups. The techniques are not limited to the codon substitution process, but may be applied to systems that are specified by a state space and a transition matrix. This is a very broad class of systems that for instance include networks (where vertices constitute the state space, and where the transition matrix is defined by the network Lagrangian). The techniques may therefore be used to identify hierarchical dynamics in seemingly very different biological systems, such as protein interaction networks, genetic regulatory networks, metabolic pathways and food webs. Identifying the hierarchical structure of a system does not only increase our understanding of the system, especially if the levels are intuitively difficult to grasp, but it also enables effective coarse graining in simulations. As soon as one hierarchical level is identified, details of lower levels can be discarded if they are not of relevance when simulating the system at the new level. Due to the increasing size and complexity of current models that owe to the rapid growth of available biological data, such reductions are of particular value.

## Authors' contributions

OG conceived of the study. OG and MNJ designed and implemented the algorithms, performed the computational experiments, analyzed the results, wrote the paper, and read and approved the final manuscript.
